# EDUCATION AND PERCEIVED FUTURE NEED FOR ADL HELP

**DOI:** 10.1093/geroni/igac059.313

**Published:** 2022-12-20

**Authors:** Julia Finan

**Affiliations:** Syracuse University, Syracuse, New York, United States

## Abstract

Extant literature suggests that adults with higher education are more likely to avoid poor health outcomes and to rate their health as better than individuals with less education. The current study builds on prior research by analyzing the association between educational attainment and the perceived need for future help with activities of daily living (ADLs), often measuring disability in the literature. 2011-2014 National Health Interview Survey (NHIS) data for adults in the United States age 40 to 65 (N=55,166) were analyzed using multivariate regression analysis. Among non-Hispanic Whites, increased years of education predicted stronger anticipation of the need for future ADL assistance. For non-Hispanic Blacks, this relationship was reversed at the some-college level. Education was not predictive of perceived future ADL assistance need for all other racial-ethnic groups. Results of this study suggest education has a unique impact on anticipation of future need for ADL assistance among non-Hispanic Whites.

